# Contemporary results of transcatheter mitral valve procedures: bi-centric retrospective analysis

**DOI:** 10.1186/s43044-022-00257-x

**Published:** 2022-03-28

**Authors:** Tamer Owais, Mohammad El Garhy, Sebastian Elvinger, Eva Harmel, Tatiana Maria Sequeria Gross, Harald Lapp, Thomas Kuntze, Wolfgang Von Scheidt, Evaldas Girdauskas, Mahmoud Al-Jassem, Philipp Lauten

**Affiliations:** 1 Department of Cardiac Surgery, University Hospital Augsburg, Stenglin Street 2, 86156 Augsburg, Germany; 2 Department of Cardiothoracic Surgery, Cairo University, Giza City, Egypt; 3 Heart Centre, Zentralklinik Bad Berka, Bad Berka, Germany; 4 Department of Cardiology, Minia University, Minya, Egypt; 5 Department of Cardiology, University Hospital Augsburg, Augsburg, Germany

**Keywords:** Minimally invasive, Mitral valve, TMVIR, TMVIV, Mitral regurgitation, Mitral stenosis, Transapical, Transseptal

## Abstract

**Background:**

Transcatheter mitral valve-in-valve (TMVIV) or valve-in-ring (TMVIR) replacement offer an alternative therapy for high risk patients. We aimed to highlight the operative and postoperative results of TMVIV and TMVIR procedures.

**Results:**

We included all patients underwent TMVIV and TMVIR procedures between 2017 and 2020 at two heart centers in Germany. We included a total of 36 high risk patients in our study where 12 received TMVIV and 24 received TMVIR. All patients underwent TMVIV or TMVIR with Edwards Sapien XT or S3 transcatheter valves (Edwards Lifesciences). The mean age was 79 (75–83 years old). The median (IQR) preoperative STS score was 9 (7–13)% and EuroSCORE II was 14.5% (12–16). The majority of our patients were operated via transapical approach (*n* = 26) and the minority via transseptal approach (*n* = 10). Out of our records, none of our patients required reopening for bleeding or any other surgical complications. None of our patients required reintervention during the 6 months follow-up period. One mortality was recorded on fifth postoperative day due to low cardiac output syndrome (obviously because of LVOT obstruction by the anterior mitral leaflet). The average blood loss was 200 ml in the first 24 h in patients underwent transapical approach. Average operative time was 93 min and all patients were immediately extubated after the procedure in the operating room (even the patient with echocardiographically documented LVOT obstruction who died on the fifth postoperative day). Length of Intensive Care Unit stay was 2 ± 1.2 days and length of hospital stay was 4.1 ± 1.2 days. In the follow up period, echocardiograms showed normal prosthetic valve function with low transvalvular gradients, no LVOT obstruction in TMVIR cases and no evidence of valve migration or thrombosis (except in one patient). Concerning 6 months readmission, it was recorded in 2 patients due to right sided heart failure symptoms due to preexisting high degree of tricuspid valve regurge which did not disappear or even decrease after the operation and the other patient due to gastrointestinal bleeding.

**Conclusions:**

TMVIV and TMVIR offer an efficient, safe and less invasive alternative in high surgical risk patients.

## Background

Despite the progressive rise in the number of cases in need of repeated mitral surgery and improved outcomes, redo mitral valve surgery remains particularly a high risk procedure in high risk surgical candidates and can be associated with high morbidity and mortality. Multiple reports state a 30-day mortality ranging between 6.3 and 15% for elective cases and 17.8% for emergency cases [1, 2]. Redo mitral valve replacement (MVR) is a complex procedure with the technical challenge of reopening a previously operated chest. The incidence of Redo mitral valve surgery in the first 10 years is 35% [1, 2]. As well, TMVR pose several challenges including lack of direct visualization and lack of direct fixation with sutures which requires accurate pre-procedural planning. Transcatheter mitral valve replacement (TMVR) is rapidly developing as a safe alternative to surgery for patients with degenerated mitral bioprostheses and failed repairs with annuloplasty rings. MViV for high surgical risk patients was approved by the Food and Drug Administration in the United States on June 5, 2017, while MViR and ViMAC remain off-label. TMVR remain in the early stages of clinical experience in high-risk patient population. However, there are limited data from registries suggest that transcatheter mitral valve-in-valve (MViV) and mitral valve-in-ring (MViR) are feasible with reasonable outcomes in high surgical risk patients [3–5]. Long et al. showed in single-center study, which included 24 high risk patients, that TMVIV and TMVIR is safe and associated with low procedural complications, mortality, and readmission rates for congestive heart failure at 30 days, 180 days, and 1 year were very low in this high-risk cohort [4]. Hu et al. showed in a systematic literature review of a total of 245 patients (172 patients who underwent TMVIV surgery and 73 patients who underwent TMVIR surgery, 55.2% TA aaproach) that the success rate was 93.5%. The mortality rates at discharge, 30 days, and 6 months were 5.7%, 8.1%, and 23.4%, respectively [3]. Simonato et al. showed, in the largest TMVR trial up till now that Mitral (857 ViV, 222 ViR), that ViV has acceptable safety and clinical outcomes in a select group of high-risk patients. However, Mitral ViR was associated with lower success rates, lower survival and higher rates of post-procedural MR. Significant residual MS and/or MR were not infrequent after mitral ViV and ViR procedures and were both associated with a need for repeat valve replacement. Strategies should be explored to prevent residual MR and MS in order to prolong device durability and patient symptom-free survival after ViV and ViR procedures [5]. In this study, we sought to retrospectively analyze the feasibility and safety of these procedures (TMVIV and TMVIR) in 2 heart centers in Germany which are respecting and following the same rules and protocols.

## Methods

We conducted a retrospective analysis for all TMVIV and TMVIR procedures (*n*: 36) through Transseptal (*n*: 10) and Transapical (*n*: 26) approach between 2012 and 2021. The 2 involved participating centers used standardized preoperative data collection, operative protocols and techniques as well as postoperative variables with clinical outcomes from the index hospitalization until 6 months follow up.

**Fig. 1:**
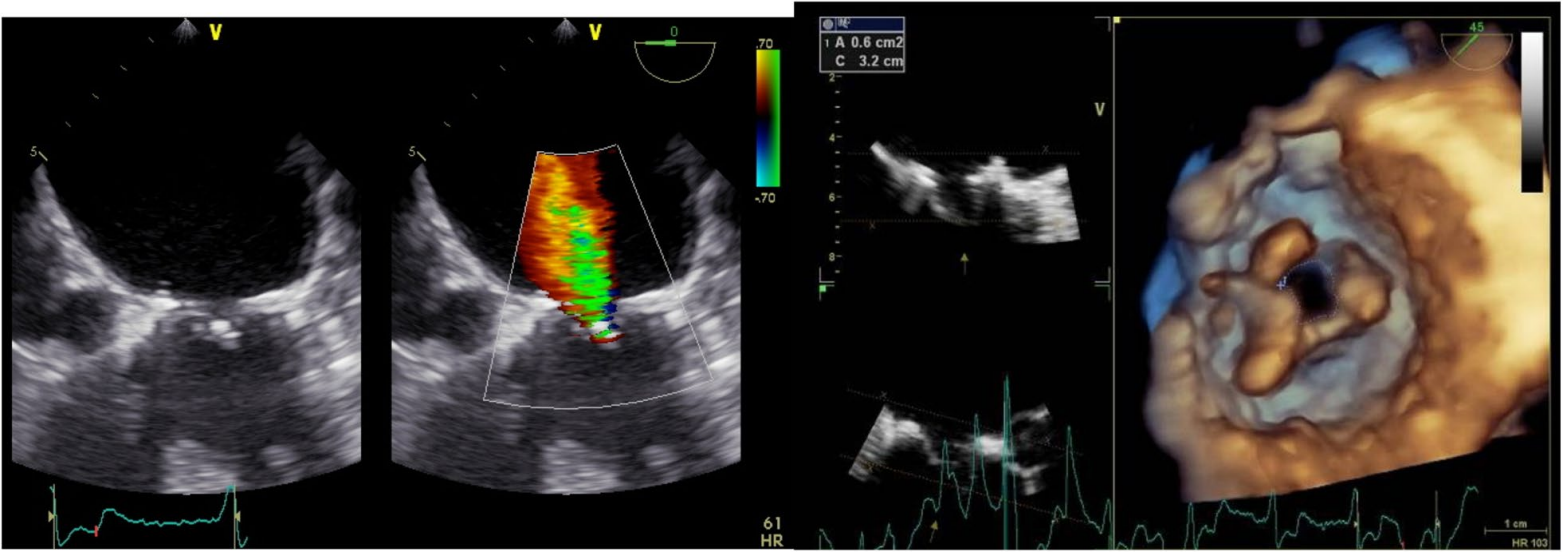
preoperative TEE showed comined severe mitral stenosis (MVA 0.6 cm^2^) and moderate valvular mitral regurge. TEE: transesophageal echokardiography, MVA: mitral valve area

**Fig. 2:**
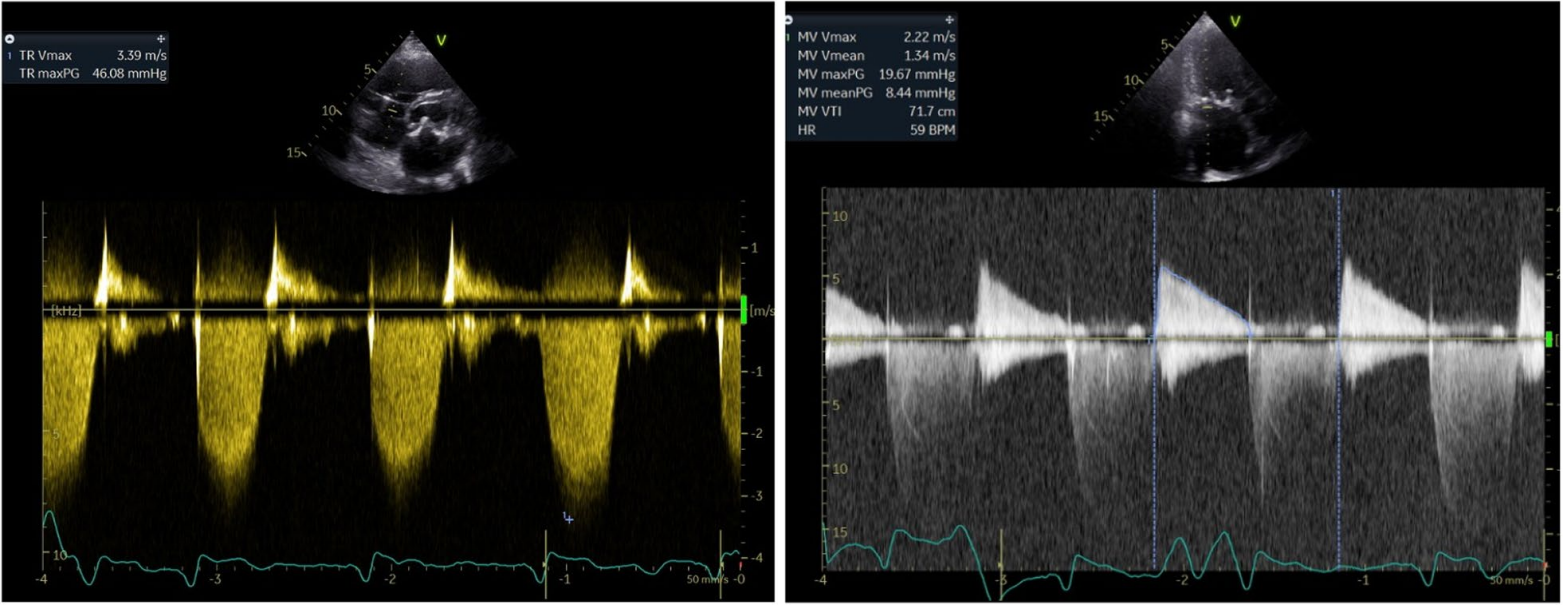
hemodynamic assessment of the valvular lesion with TTE showed mean gradient over the prthesis of 8 mmHg and moderate pulmonary hypertension (45 mmHG1,8, flow + CVP). TTE: transthoracic echocardiography; CVP: central venous pressure

**Fig. 3:**
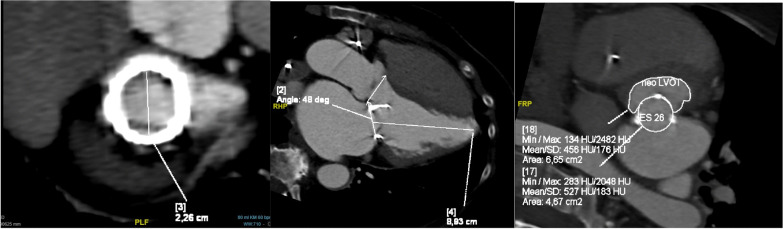
CT assessment of (1) internal diameter of the prosthesis, (2) aortomitral angel of 132°, (3) neo LVOT area of 6.6 cm^2^. LVOT: left ventricular out flow tract

We demonstrated all demographical data, including age, gender, Diabetes type II, Carotid stenosis, Chronic renal insufficiency, Chronic obstructive pulmonary disease, previous heart surgery, ejection fraction, pulmonary hypertension, NYHA class (New York Heart Association), EuroSCORE II, STS Score and atrial fibrillation. Preoperatively, all patients were discussed in a multidisciplinary structural heart team, consisting of structural heart cardiologists, cardiothoracic surgeon (who worked in the 2 centers), and anesthesiologist. Routinely, preoperative cardiac catheterization, transthoracic echocardiogram (TTE) (Figs. 1, 2), lung functions tests, laboratory investigations and cardiac computed tomography (CT) scans were done. Special consideration was taken to determine the neo left ventricular outflow tract (LVOT), aortomitral angel and the internal diameter of the prosthesis or ring (Fig. 3). Valve size was decided according to the the Valve in Valve (Mitral) app developed by Vinayak Bhapat, MD (UBQO Limited).

All operations took place in the hybrid room with a heart lung machine in stand-by and under general anesthesia. Perioperative data were collected including, previously implanted bioprosthesis or annular ring, operative time, need for inotropes, transesophageal echocardiogram (TEE) (for determining residual mitral regurgitation greater than 1 + central or paravalvular leaks, transvalvular gradients and leaflets mobility); procedural mortality, competent vascular or transapical access and need for emergency surgery or re-intervention (eg: significant right to-left interatrial shunt, LVOT obstruction, femoral A V fistula, or tamponade).

**Fig. 4:**
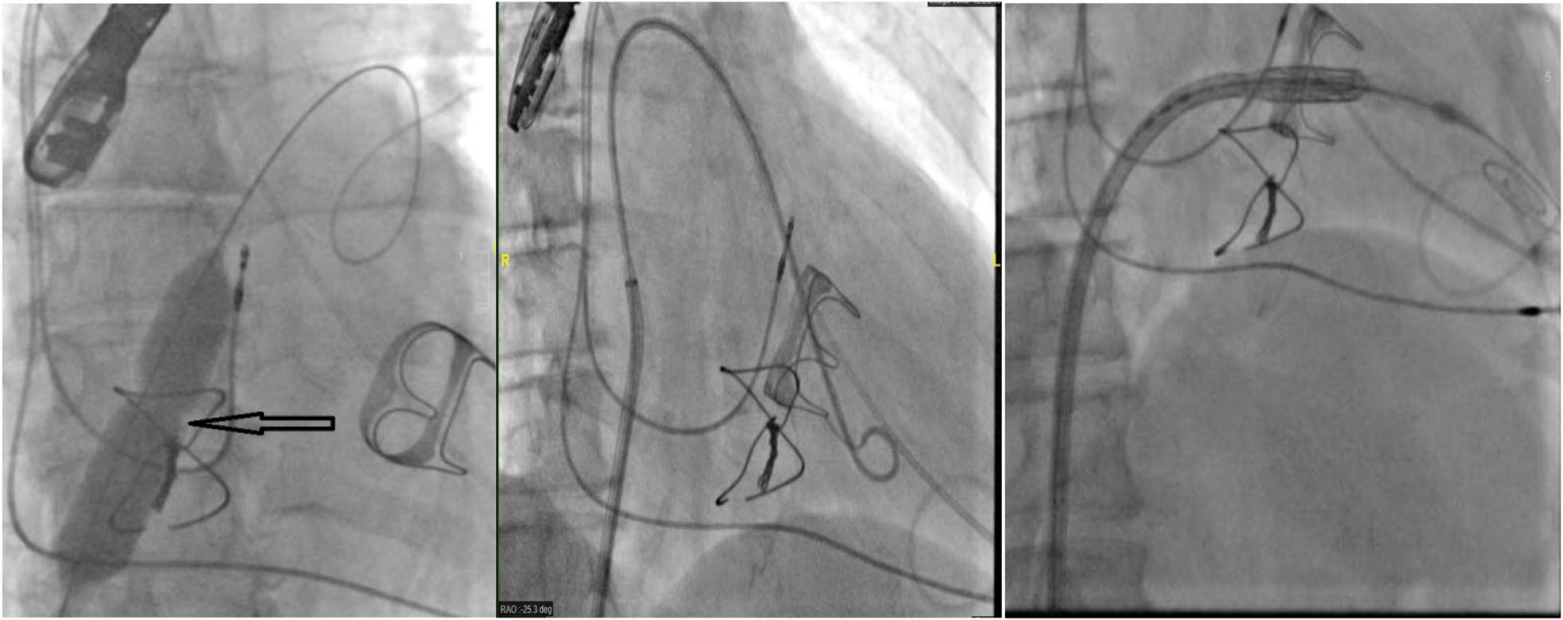
important steps of the intervention: (1) dilatation of septum according with appropriate ballon size according to the device size and the anatomy of IAS, (2) the passage of pigtail into LV using steerable catheter or by advancing the catheter in the direction of LA roof to make a large loop in LA, (3) slowly withdrawal of the pigtail to get rid of this large loop in LA and then change to stiff preshaped wire

**Fig. 5:**
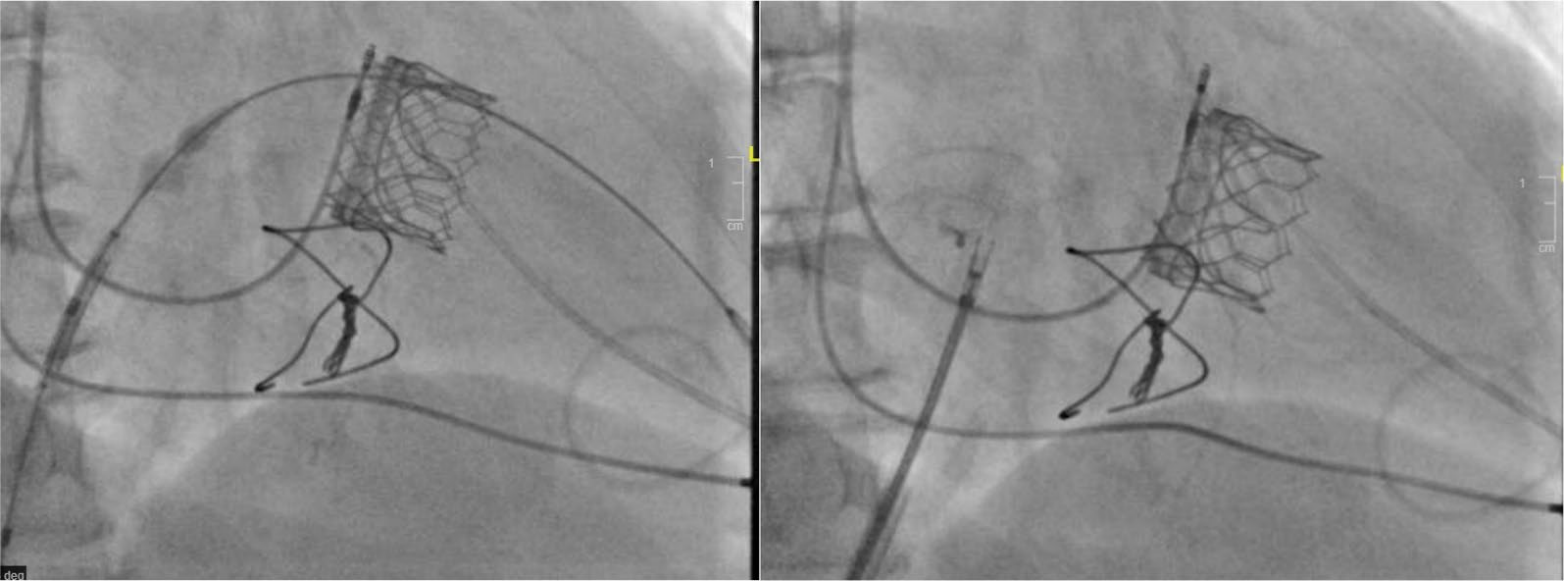
(1) proper positioning of valve is curcial to avoid valve migration, (2) after successful valve implantation assessment of the iatrogenic ASD is mandatory to decide the need of ASD closure in case of hemodynamic significance as in this case

**Fig. 6:**
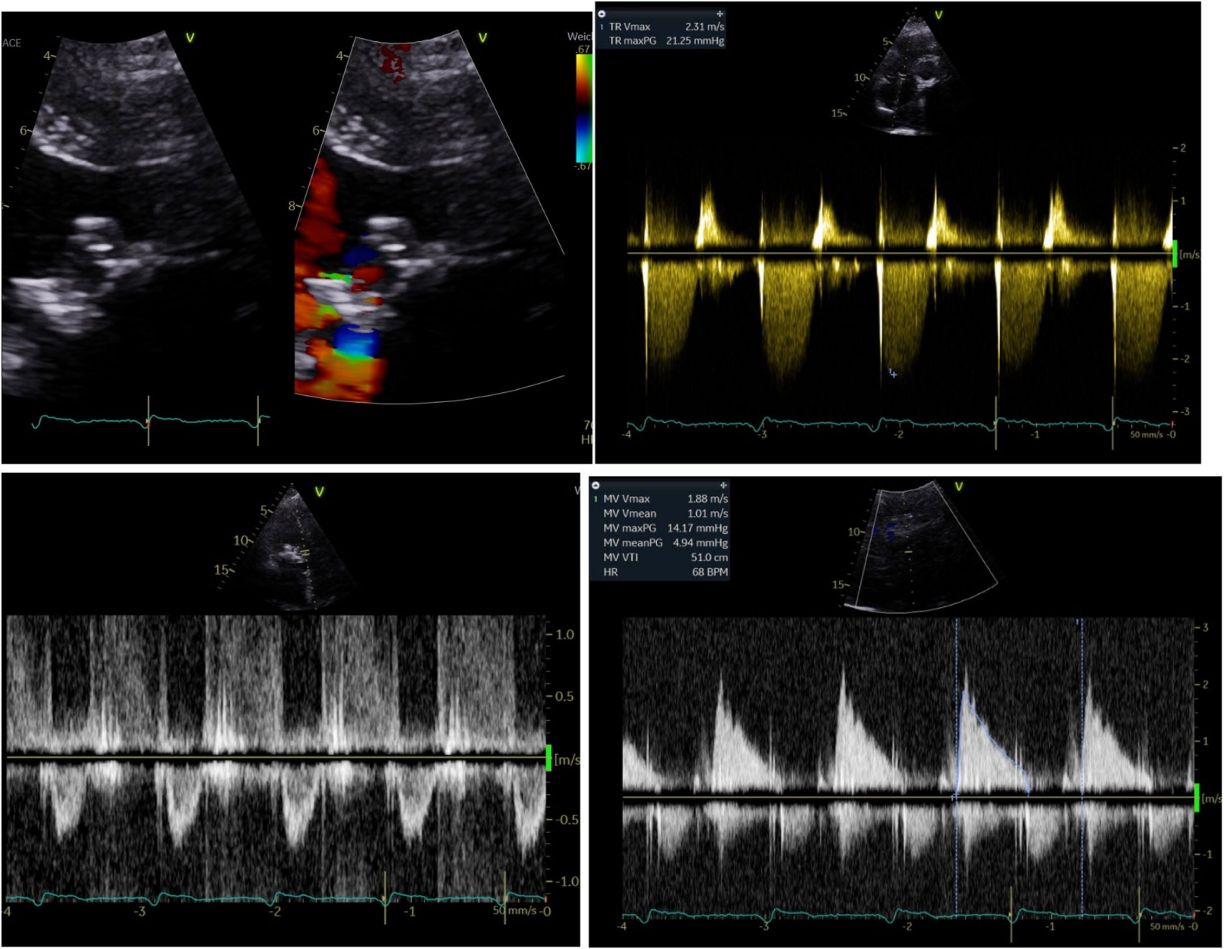
6-months echokardiographisch assessment showed marked only trivial paravalvular regurge (1), significant reduction of the pulmonary artery pressure from 45 mmHg + CVP to 26 mmHg + CVP (2), no LVOT obstruction (3), mean Gradient < 5 mmHg (4). Die war. Der Urin

1. Our routine practice in transseptal cases entailed preclose with two ProGlide devices (Abbott Vascular, Santa Clara, California) in the right common femoral vein and the Edwards Sheath was introduced after administration of Heparin 100 U/kg) (ACT > 300 s).
2. Transseptal puncture was performed under TEE guidance and then balloon dilated with 14 Mustang (Boston scientific) in most of cases but if in patients with history of surgical ASD closure with path and also if they will receive 29 mm ES valve we dilated with 18 mm ballon.
3. Pacing was done using the LV wire.
4. An 8.5-F medium curve Agilis sheath (St. Jude Medical, St. Paul, Minnesota) was placed in the left atrium and directed over the mitral valve or ring to ease valve passage with a straight wire. Alternative to this approach is the use of pigtail catheter with Terumo: by advancing the catheter in the direction of LA roof to make a large loop in LA and then slowly withdrawal of the pigtail to get rid of this large loop in LA and then change to stiff preshaped wire (Fig. 4).
5. A SAFARI wire was routinely used to add for maximum stability of the catheter valve during introduction and implantation.
6. Care was taken in valve in ring cases not to oversize the catheter valve in order not to deform the oval shaped annuloplasty ring into a rounded shaped one and thus leads to para-ring leakage. On the other hand we intended to oversize with 2 ml in valve in valve cases to achieve a funnel shaped ventricular end of the catheter valve (Fig. 5).
7. In the postoperative phase until 6 months follow up, we collected the data of the discharge echo and at 6 months as well (transmitral gradient, Valve migration, and leaflet mobility, leaflet dysfunction due to thrombosis, Ejection fraction and LVOT morphology) (Fig. 6). In addition, we searched for hemolytic anemia and cerebrovascular strokes in the follow up data base. All patients were interviewed on phone calls and evaluated for quality of life and history or readmission as well. It’s worth noting that all patients were given warfarin until 6 months routinely which coincides accidently with our follow up time frame. In our cohort, we had no patients with contraindication to warfarin. This retrospective quality review study was previously approved in both institutes.

## Statistical analysis

Continuous variables were tested for normality of distribution by using the Shapiro-Wilk test. Normally distributed variables were expressed as mean ± standard deviation. For non-normally distributed variables, the median and inter-quartile range (IQR) were calculated. Categorical variables were expressed in number and percentage. Statistical analyses were performed with SPSS (version 24.0; IBM Corporation, Armonk, NY).

## Results

### Preoperative and demographic data

Baseline demographic and echocardiographic data are shown in Table 1, the mean age was 79 (75–83 years), 15 females and median preoperative STS score was 9 (7–13)% and EuroSCORE II was 14.5% (12–16). We excluded no patients according to preimplanted valve or ring. NYHA class III or more was met in 31 patients while 4 patients were NYHA class II but with rash progressive rise in the transmitral valvular gradient in the last average 2 months. One patient was mentally retarded and could not be accurately examined for symptoms, only through asking his mother who stated that he started to show dyspnea at rest and while eating. Despite the lower ejection fraction and higher degree of regurge in patients with previously implanted ring, yet the results remain statistically non-significant.

**Table 1:** Baseline characteristics of study patients

	36 patients
Age (years)	79 (75–83)
Female *n* (%)	15 (41%)
COPD *n* (%)	6 (16.6%)
CRF > II *n* (%)	3 (8.3%)
NYHA III- IV *n* (%)	31 (86%)
Previous myocardial infarction *n* (%)	8 (32%)
pHTN (%)	25 (63%)
BMI > 30 *n* (%)	10 (27%)
Creatinine [mg/dl] (preoperative)	1 ± 0.2
EuroSCORE II (median IQR)	14.5 (12–16)
STS score (median IQR)	9 (7–13%)

COPD, chronic obstructive pulmonary disease; CRF, chronic renal failure; n, number; SD, standard deviation; NYHA, New York Heart Association; pHTN, pulmonary hypertension; BMI, body mass index

### Procedural outcomes

Table 2 shows all operative variables. Of the 36 patients who underwent TMVR, 26 patients were operated upon via transapical approach and the rest 10 patients via transseptal approach. 70 % had a previously implanted bioprosthesis. We converted from transapical to transseptal approach in february 2017 after building up enough experience in the echocardiographic septal puncture throughout our mitral clip program. We had no conversion between the 2 approaches due to either failed retrograde mitral valve passage or failed transseptal puncture. A 29-mm Edwards life science valve was used in 22 patients while the 26-mm Edwards valve was used in the rest 16 patients and mostly in cases with previously implanted ring. Concerning the occurrence of LVOT obstruction, we could document one patient intraoperatively with drastic increase in the intracavitary gradient through the anterior mitral leaflet after the implantation of a 26 mm Edwards valve in an Edwards physio ring 30 mm. Fortunately, no progressive rise in the LVOT gradient was noted in the serial echocardiographic assessment of other patients through the follow up period and thus no further septal ablation was required. We recorded no incidence of severe paravalvular or para-ring leakage requiring intervention immediately after valve implantation. Only one patient showed moderate paravalvular leakage. It is worth noting that 2 patients had previously para-ring leakage documented through preoperative echo after excluding endocarditis and ring dehiscence. This para-ring leakage did worsen in one patient 3 months after valve in ring implantation. Mean postoperative gradient was (3.1 ± 2.1 mm Hg) and postoperative ejection fraction was (54.1 ± 15.12%).

**Table 2:** Operative results and postoperative endpoints

*N* = 36 patients	
TS approach	10 (27%)
Edwards Sapien	36
26 mm	16 (56%)
29 mm	22 (16%)
Implantation success	0
Valve embolization	0
Conversion to sternotomy	0
LVOT Obstruction	1 (2.7%)
Pericardial tamponade	0
Intraaortic balloon pump	0
Emergency cardiopulmonary bypass	0
Extubation in OR	36 (100%)
≥ post. moderate PVL	1 (2.7%)
mPG postop	3.1 ± 2.1
6-months mortality	1 (2.7%)
AKI	1 (2.7%)

N, number; SD, standard deviation; AKI, acute kidney injury; PVL, paravalvular leak; OR, operation theater; mPG, mean pressure gradient, TS, transseptal

### Postoperative and follow up outcomes

None of our patients needed any kind of mechanical support as ECMO or Impella or even operated upon under emergency conditions. All causes of mortality were also noted. The average blood loss was 200 ml in the first 24 h (no blood loss since we began with transseptal approach in 2017) and average operative time was 46 ± 9.2 min (with no significant difference between the 2 approaches or even between the previously implanted valve and ring). There was no significant difference in transvalvular mitral valve gradient between cases with prior valve or ring. Length of Intensive Care Unit stay was 2 ± 1.2 days (longer in TMVIR than TMVIV) and length of hospital stay was 4.1 ± 1.2 days (longer in TMVIR than TMVIV). The patient with intaoperatively documented LVOT obstruction was extubated in the operating room but reintubated after 12 h duo to progressive Low cardiac output syndrome and increased inotropic support and hence stayed in the ICU until she passed away on the fifth day. Anemia was noticed in 2 patients on the first postoperative day, probably hemolytic type (hematuria was noticed). The 2 patients were on warfarin preoperatively and INR was in therapeutic range and one of them reported accidental hematuria in the last 6 months prior to surgery. No acute renal failure or myocardial infarction or cerebrovascular insult or new onset atrial fibrillation was recorded in our postoperative course. During the follow up 6 months period, we recorded one readmission due to right sided heart failure because of preexisting Tricuspid regurge and one readmission due to gastrointestinal bleeding not requiring intervention but only transfusion (This patient had preoperatively intestinal polyps and was on warfarin therapy). Concerning paravalvular or ring leakage, as previously mentioned, it was noted in 2 patients (existed preoperatively). One patient had stable leakage grade throughout 4 months follow up but unfortunately we lost track to follow up the patient until 6 months. The other patient had progressive leakage until reaching severe degree of mitral regurge at 3 months follow up which was not amenable neither for intervention nor for surgery. This patient had recurrent mild symptoms of decompensation and was always successfully recompensated by optimizing the heart failure medications. It’s worth noting that this patient received an Edwards sapien 3 26 mm in a Medtronic Profile 3D 32 mm ring, which is known to be a complete rigid saddle shaped ring which lead to deformation of the Edwards valve resulting in paravalvular leakage. One mortality was recorded on fifth postoperative day due to low cardiac output syndrome (obviously because of LVOT obstruction by the anterior mitral leaflet after the implantation of a 26 mm Edwards valve in an Edwards physio ring 30 mm). In this patient, the length of the anterior mitral leaflet was 32 mm and the diameter of the subannular LVOT was 22 mm, which denotes a high incidence of LVOT obstruction. We discussed the surgical option as by emergency ECMO implantation then sternotomy and resection of the anterior mitral leaflet through an aortotomy. But unfortunately, the patient worsened dramatically and quickly in cardiogenic shock with multi-organ failure and hence our surgical plan could not be implemented. One patient had an echocardiographic finding after 4 months denoting minute atrial valve migration of 2 mm, where we were not able to perform a CT for the patient due to massively poor general condition and logistic difficulty in transport. This patient was operated upon in our early phase before establishing a sensational learning curve and received Edwards XT 29 mm in Mosaic 31 mm and probably the primary implantation was higher than supposed (after retrospectively examining our implantation films). Our TTE results for 36 patients showed Mean postoperative gradient was (3.1 ± 2.9 mm Hg), postoperative ejection fraction was (54.1 ± 15.12%) and mean pulmonary artery pressure of 51.5 ± 11.9 mm Hg, with stable mean valve area (MVA). Our access outcomes were statistically nonsignificant concerning bleeding, hematoma or AV Fistula in the transseptal group and concerning wound dehiscence in the transapical group. We have one patient showed surgical subcutaneous emphysema after a transapical approach which did not require prolonged intensive therapy but required an extra pleural drainage which lasted for 2 days and due to resolution of emphysema, we removed the pleural tube on the third postoperative day.

## Discussion

In this Bi-Centric retrospective study, we could conclude that TMVIV/TMVIR can be a safe and effective therapeutic modality in patients with prior mitral valve surgery. Our statistical data could not be compared with these of published recently in January 2021 because of the size of cohort group (857 patients) and the multicentric design [5]. Our cohort group is smaller and bi-centric. This would add for the weight of our study in one aspect which is the common standardized pre, intra and postoperative protocols, since the same surgeon was responsible for these procedures in the 2 hospitals and he implemented the same management protocols as a routine.

In comparison to Long et al., who published his paper in 2018, we have contrary study groups [4]. We have more patients operated in transaapical approach than in transseptal approach. This could be explained through the fact that we have a reasonable experience in transapical approaches because we operated up to 50% of our TAVI patients until 2012 as transapical. That’s why we could operate our transcatheter mitral valve procedures only in transapical approach until our Mitraclip team was able to build up reasonable experience in septal puncture by 2017. This explains why we did not have any conversion rate due to failure to retrograde passing the valve. Our retrograde passing protocol was as follows; at first, a straight Teflon wire was used and if failed, we use Terumo straight wire (sometimes under rapid pacing). We had to use the Agillis sheath in one patient as a third option.

The reason that we have the majority of our patients are with prior implanted mitral rings is that we are considered as a referral center in East Germany for Minimmally invasive mitral valve repair (Ring + artificial loops). As a matter of fact, the TMVIR cases are more challenging and more complex cases when compared to TMVIV cases. In TMVIR cases, we have to study the detailed anatomy of the LVOT and Anterior mitral leaflet to avoid LVOT obstruction.

Concerning the incidence of LVOT obstruction, we reported one patient with documented intraoperative LVOT obstruction who died on the fifth postoperative day. Long et al series documented 2 cases out of 24 patients [4]. We could admit that our postoperative diagnostic tool for LVOT obstruction (Only Echocardiography) was less accurate than what was implemented in Ashleigh long series (CT). This means we could have missed diagnosing this phenomena in our cohort series and if we had done CT routinely, we could have found out more patients with this complication.

Hu et al reported >12% of the patients more than mild mitral regurge (Paravalvular) postoperatively [3]. We reported only one patient with moderate paravalvular leakage in our series with prior mitral annuloplasty ring. This could be explained that all of our patients had previously a semi rigid complete ring (physio 2 ring) which is known to have lower chance of paravalvular leak because it will be changed in to a circular ring by the implanted Edwards sapien TAVI valve.

Our average blood loss is significantly more than what is reported in Ashleigh series. This could be explained may be through the fact that we never stop the anticoagulation medications preoperatively. On the other hand, we lie far beyond the drainage loss reported by Mehaffey HJ who published the results of redo surgical mitral valve surgery and published in 2018 [6]. This adds to our philosophy that Transcatheter approaches in such cases could be safer concerning bleeding. Length of ICU and hospital stay in our study coincides with most of the paper published in this domain. In most of the centers, these procedures are whether done under local anesthesia or general anesthesia with light sedation where the patient is extubated immediately on the operating table. Anemia postoperatively was not a studied parameter in many of the studies in this domain. In our study, we reported 2 patients (5.5%) with hemolytic anemia postoperatively who were on warfarin therapy preoperatively. Our results coincides with Coylewright M who reported anemia in 6.1% of his study population [7]. We coincide together in not stopping the anticoagulants preoperatively as well, which may explain our coincidence. Our results concerning mean transvalvular gradient postoperatively, showed lower gradients in comparison to Eleid MF [8]. This could be explained throughout that most of our patients were with prior implanted semi rigid complete ring and we intended to oversize in most of our patients. Fortunately, despite oversizing, we found out no patients with new severe paravalvular leakage due to ring deformation or dehiscence. Our total operative time was shorter than Long et al [4]. This is probably because of the common same operator in all of the 36 cases (routine maintained) or may be because we did not perform valvoplasty in any of our cases or due to the routine usage of Agilis sheath for valve passage in transseptal cases which might save few minutes.

## Study limitations

The retrospective observational design does not allow for a proper comparison between either surgical or conservative strategies. Besides, small sample size and limited follow up to 6 months are clear limiting factors in this study. No much attention was taken to assess the Neo LVOT morphology in details due to lack of our experience in this domain.

## Conclusions

Mitral valve interventions in the form of TMVIV and TMVIR are efficient, safe and less invasive in candidates with high surgical risk for conventional surgery. Periprocedural outcomes were satisfying concerning operative time and postoperative bleeding and intubation time. At 30 days and 6 months follow up, all patients experienced stable echocardiogram findings concerning the valve dynamics and improvement of life style.

## Data Availability

All data are available on request at the Department of Cardiology, Zentralklinik Bad Berka, Germany.

## Abbreviations

ASD: Atrial septum defect
CT: Computer tomography
IQR: Interquartile range
LVOT: Left ventricular outflow track
MR: Mitral regurge
MS: Mitral stenosis
SD: Standard deviation
TMVIV: Transcatheter mitral valve-in-valve
TMVIR: Transcatheter mitral valve-in-ring
TTE: Transthoracic echocardiogram
TEE: Transesophageal echocardiogram

## References

[1] Onorati F, Perrotti A, Reichart D (2016). Surgical factors and complications affecting hospital outcome in redo mitral surgery: insights from a multicentre experience. Eur J Cardiothorac Surg.

[2] Vohra HA, Whistance RN, Roubelakis A (2012). Outcome after redo-mitral valve replacement in adult patients: a 10-year single-centre experience. Interact Cardiovasc Thorac Surg.

[3] Hu J, Chen Y, Cheng S (2018). Transcatheter mitral valve implantation for degenerated mitral bioprostheses or failed surgical annuloplasty rings: a systematic review and meta-analysis. J Card Surg.

[4] Long A, Mahoney P (2018). Transcatheter mitral valve-in-valve and valve-in-ring replacement in high-risk surgical patients: feasibility, safety, and longitudinal outcomes in a single-center experience. J Invasive Cardiol.

[5] Simonato M, Whisenant B, Ribeiro HB (2021). Transcatheter mitral valve replacement after surgical repair or replacement: comprehensive midterm evaluation of valve-in-valve and valve-in-ring implantation from the VIVID registry. Circulation.

[6] Mehaffey HJ, Hawkins RB, Schubert S (2018). Contemporary outcomes in reoperative mitral valve surgery. Heart.

[7] Coylewright M, Cabalka AK, Malouf JA (2015). Percutaneous mitral valve replacement using a transvenous, transseptal approach: transvenous mitral valve replacement. JACC Cardiovasc Interv.

[8] Eleid MF, Whisenant BK, Cabalka AK (2017). Early outcomes of percutaneous transvenous transseptal transcatheter valve implantation in failed bioprosthetic mitral valves, ring annuloplasty, and severe mitral annular calcification. JACC Cardiovasc Interv.

